# Monocytes Expand with Immune Dysregulation and Is Associated with Insulin Resistance in Older Individuals with Chronic HIV

**DOI:** 10.1371/journal.pone.0090330

**Published:** 2014-02-27

**Authors:** Cecilia M. Shikuma, Dominic C. Chow, Louie Mar A. Gangcuangco, Guangxiang Zhang, Sheila M. Keating, Philip J. Norris, Todd B. Seto, Nisha Parikh, Kalpana J. Kallianpur, Beau K. Nakamoto, Lorna S. Nagamine, Lishomwa C. Ndhlovu, Jason D. Barbour

**Affiliations:** 1 Hawaii Center for AIDS, University of Hawaii, Honolulu, Hawaii, United States of America; 2 Blood Systems Research Institute, San Francisco, California, United States of America; 3 University of California San Francisco, San Francisco, California, United States of America; 4 Queen’s Medical Center, Honolulu, Hawaii, United States of America; 5 Straub Clinic and Hospital, Honolulu, Hawaii, United States of America; New York University, United States of America

## Abstract

**Background:**

Rates of insulin resistance are increased in HIV-infected patients on stable antiretroviral therapy (ART). Such increase may partially be due to HIV-induced immune dysregulation involving monocytes (MO) and its subsets.

**Materials and Methods:**

Cross-sectional analysis of 141 HIV-infected subjects age ≥ 40 years on stable ART. Homeostatic model assessment–insulin resistance (HOMA-IR) and rates of metabolic syndrome were calculated. Subjects were classified by fasting glucose and oral glucose tolerance test (OGTT) into clinical diabetes categories. Multi-parametric flow cytometry was used to determine MO subset percentages: [classical (CD14^++^CD16^−^), intermediate (CD14^++^CD16^+^), non-classical (CD14^low/+^CD16^++^), and a recently identified fourth (CD14^low/+^CD16^−^) ‘transitional’ MO subset] and percentage of activated (CD38^+^HLA-DR^+^) CD8 T cells. Absolute levels of cells were calculated using clinical CBC and T cell subset data. Multiple plasma soluble biomarkers were assessed by Luminex technology.

**Results:**

Median age 50 years, CD4 count (percent) 505 cells/µL (29%), and 89% male. Total MO (r = −0.23, p = 0.006) and classical and non-classical MO subsets correlated negatively with CD4 percent. No correlations were seen with CD4 count as absolute values. Log-total MO and log-classical MO predicted HOMA-IR independently of HIV immuno-virologic and diabetes risk factors (β = 0.42, p = 0.02 and β = 0.35, p = 0.02, respectively) and were increased in subjects with metabolic syndrome (p = 0.03 and p = 0.05 respectively). Total and/or subset MO levels correlated with multiple soluble plasma biomarkers including CRP, IL-6, MMP-9, MPO, SAA, SAP and tPAI-1, with tPAI-1 independently predicting HOMA-IR (β = 0.74, p<0.001).

**Conclusions:**

MO levels increase with worsening HIV immune dysregulation as assessed by CD4 percent. CD4 percent may provide additional information about MO and metabolic risk in this population beyond absolute values. MO, and specifically classical MO, may contribute to insulin resistance and metabolic syndrome during chronic HIV infection. Multiple soluble plasma biomarkers including tPAI-1 increase with increase in MO. Levels of tPAI-1 independently predict the development of insulin resistance.

## Introduction

Abnormalities of glucose metabolism occur commonly among HIV-infected patients on stable antiretroviral therapy (ART) [1] and may contribute to increased rates of cardio-metabolic complications. The etiologies for the higher prevalence of insulin resistance and diabetes among ART-treated individuals with chronic HIV are likely multifactorial including the possible effects of various antiretroviral medications, presence of lipodystrophy, co-infection with hepatitis C virus (HCV), higher rates of hypogonadism, and higher rates of traditional risk factors in some HIV populations such as obesity and physical inactivity [1]. HIV-induced immune dysregulation and inflammation may also play a role in the increased prevalence of insulin resistance and diabetes. Low CD4 count is an independent risk factor for altered insulin sensitivity [2]. Higher systemic levels of C-reactive protein (CRP), and soluble receptors of tumor necrosis factor-alpha (TNF-α), sTNF1 and sTNF2, accompany incident diabetes after initiation of HIV antiretroviral therapy [3].

While studies of HIV-induced immune activation have traditionally focused on the role of CD8 T cells, there is increasing interest in the role that MO may play in non-infectious complications seen among individuals with chronic HIV. MO originate in the bone marrow and circulate in the peripheral blood for 1−3 days before differentiating into macrophages within various tissues. MO are a heterogeneous population of cells, and are classified by international consensus into several subsets on the basis of their CD14 and CD16 surface expression: classical MO lacking CD16 expression (CD14^++^CD16^−^) and those expressing CD16 comprised of intermediate (CD14^++^CD16^+^) and non-classical (CD14^low/+^CD16^++^) MO [4]. We have recently published data reporting the identification of a fourth MO subset which we have termed ‘transitional’ MO. This transitional MO subset is characterized by reduced but still detectable levels of CD14 (CD14^+^CD16^−^) and is associated within our cohort of HIV-infected individuals on stable ART with increases in carotid intima-media thickness, a well-established marker of arterial injury [5], [6]. Classical MO account for roughly 80−90% of circulating MO in normal healthy individuals. This population has been reported to increase in acute inflammation and to be rapidly recruited to sites of infection [7], [8], [9]. The CD16-containing population increases with aging and chronic inflammatory disorders, and compared to classical MO, shows higher expression of pro-inflammatory cytokines, higher potency in antigen presentation, and is more permissive for productive HIV infection [10], [11], [12]. The CD16-containing MO subset expands with HIV infection and is increased in subjects with HIV-associated neurocognitive disorders [13], [14]. An increase in non-classical MO has been reported to correlate with HIV disease progression in ART-naïve subjects [15]. Increases in both non-classical and intermediate MO subsets similar in pattern to those in HIV-uninfected subjects with acute coronary syndrome have been reported in HIV-infected individuals with uncontrolled HIV disease [14]. In the general population, increases in circulating MO have been observed in diabetic subjects compared to nondiabetic controls [16], [17], [18]. CD16-bearing MO subsets have been reported to be increased in patients with type 2 diabetes and in particular in those with diabetic complications such as renal disease [16], [19]. Taken together these data indicate a potentially important role for MO populations in the pathogenesis of HIV-associated cardio-metabolic disorders.

This study sought to understand how HIV-induced immune dysregulation influences MO and its subsets, and to then assess the relationship of these MO to insulin resistance and glucose dysregulation among HIV infected individuals on stable ART. We also assessed CD8 T cell activation levels and levels of various soluble plasma biomarkers as these parameters are known to play a substantial role in HIV immune dysregulation. We found that HIV immune dysregulation, as assessed specifically by CD4 percent but not as absolute CD4 count, is associated with an increase in MO with a very modest increase in the percentage represented by non-classical MO. In turn, we found that an increase in MO, primarily accounted for by an increase in classical MO, is associated with insulin resistance independent of HIV immuno-virologic and traditional diabetes risk factors and with metabolic syndrome. We further found that multiple positive correlations exist between numbers of total MO and/or its subsets and levels of various soluble plasma biomarkers including CRP, interleukin-6 (IL-6), matrix metallopeptidase-9 (MMP-9), myeloperoxidase (MPO), serum amyloid A (SAA), serum amyloid P component (SAP) and tissue plasminogen activator inhibitor-1 (tPAI-1). Levels of tPAI-1 increase as CD4 percent decreases and predict insulin resistance in an adjusted model.

## Materials and Methods

### Subjects and Study Design

We analyzed entry data of participants enrolled into the Hawaii Aging with HIV-Cardiovascular (HAHC-CVD) cohort, a 5-year longitudinal study investigating the role of immune activation and mitochondrial-specific oxidative stress on the pathogenesis of cardiovascular disease among HIV-infected patients on ART. The study was approved by the Committee on Human Subjects of the University of Hawaii and written informed consents were obtained from all participants.

Entry criteria required subjects to have documented HIV infection, be at least 40 years old, and be on stable ART for ≥ 6 months. Blood pressure and waist and hip measurements were obtained in triplicate and averaged. Body mass index (kg/m^2^) was calculated. CBC, T cell subsets, plasma HIV RNA assessments, chemistries and metabolic labs (glucose, insulin, total, directly measured LDL and HDL cholesterol, and triglycerides) were obtained at entry in a fasted state (nothing by mouth except water for 12 hours). Subjects were assessed for past and current tobacco use. All subjects without known history of diabetes underwent an oral glucose tolerance test (OGTT). Homeostatic Model Assessment of Insulin Resistance (HOMA-IR) was calculated using the formula: [pre-OGTT glucose (mg/dL) X pre-OGTT insulin (uIU)]/405.

Subjects were grouped by glucose dysregulation according to the American Diabetic Association Guidelines utilizing fasting glucose and results of the OGTT [20]. Impaired fasting glucose (IFG) was defined as fasting glucose between 100 and 125 mg/dL and impaired glucose tolerance (IGT) as a 2-hour OGTT glucose level between 140−199 mg/dL. Diabetes was defined as self-reported history of diabetes, use of diabetic medications, fasting blood glucose ≥ 126 mg/dL or a 2-hour OGTT glucose level >200 mg/dL. Subjects were categorized as having metabolic syndrome utilizing the parameters as proposed by the National Cholesterol Education Program’s Adult Treatment Panel III report (ATP III) [21].

### Cell Flow Cytometry

**Figure 1:**
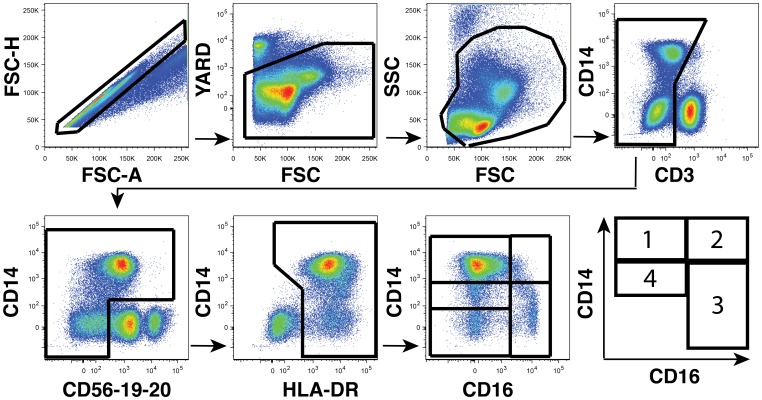
Multiparametric flow cytometry protocol utilized for identification of classical, intermediate, non-classical, and transitional monocyte subsets from peripheral blood mononuclear cells. Banked PBMC were phenotyped based on CD14 and CD16 cell surface expression into four MO subsets: [classical (CD14^++^CD16^−^); intermediate (CD14^++^CD16^+^), non-classical (CD14^+/low^CD16^++^), and transitional (CD14^+^CD16^−^)] and CD14-CD16- cells using a multiparametric panel of conjugated monoclonal antibodies that identified the HLA-DR^+^ PBMC live cell population.

Blood was separated within 30 minutes of the blood draw and peripheral blood mononuclear cells (PBMC) were isolated using Ficoll-Hypaque density gradient centrifugation (Pfizer, Inc., New York, NY). Separated PBMC and plasma were cryopreserved for future phenotypic analyses of cells and plasma cytokines. Banked PBMC were phenotyped based on CD14 and CD16 cell surface expression into 4 MO subsets [classical (CD14^++^CD16^−^), intermediate (CD14^++^CD16^+^), non-classical (CD14^+/low^CD16^++^), and transitional (CD14^+^CD16^−^)] and CD14-CD16- cells using a multiparametric panel of conjugated monoclonal antibodies that identified the HLA-DR^+^ PBMC live cell population and excluded T lymphocytes, B cells, NK cells (CD3^−^CD19^−^CD14^−^CD20^−^CD56^−^) and dead cells (aqua amine reactive dye [AARD^+^]) as shown in Figure 1. Cells are washed twice and fixed with 1% paraformaldehyde solution (PFA) before acquiring on a custom 4 laser BD Fortessa flow cytometer (BD Biosciences, San Jose, CA). Data were analyzed using Flowjo software (Treestar Inc Ashland, OR ).

The total MO count was calculated using white blood cell count (WBC) and percent MO values on the CBC conducted as part of entry evaluations on the same blood specimen as that utilized for flow cytometry. The CBC was performed by Diagnostic Laboratory Services (DLS), Inc., a local commercial Clinical Laboratory Improvement Amendments ACT (CLIA)-certified laboratory. Total numbers in each MO subset were then derived by multiplying the number of total MO by the percent of total MO representing each MO subset as derived from cell flow cytometry.

Similarly, the number of total CD8 T lymphocytes was obtained from T cell subset analyses performed by DLS as part of entry evaluations. The number of activated CD38^+^HLA-DR^+^ CD8 T cells was derived by multiplying the total number of CD8 T cells by the percentage of CD8 T cells co-expressing CD38 and HLA-DR as assessed by flow cytometry.

### Plasma Soluble Biomarkers

Testing was conducted using Milliplex Human Cardiovascular Disease panels (EMD Millipore, USA) as outlined in the manufacturer’s protocols. Soluble biomarkers assessed included MMP-9, MPO, tPAI-1, CRP, SAA, SAP, IL-1β, IL-6, IL-8, IL-10, TNF-α, soluble E-selectin (sE-selectin), soluble vascular cell adhesion molecule-1 (sVCAM-1), soluble intercellular cell adhesion molecule-1 (sICAM-1), monocyte chemoattractant protein-1 (MCP-1), vascular endothelial growth factor (VEGF), interferon-gamma (IFN-γ), and N-terminal pro-brain natriuretic peptide (NT-proBNP).

### Statistical Analyses

Demographic, clinical and immunologic information was summarized by median (quartile1, quartile3) values for continuous variables and frequency (percentage) for categorical variables. Continuous variables were first log_10_-transformed to improve normality before further analyses if their distributions were skewed. Correlations between variables were performed by Pearson correlation. The relationships between HOMA-IR and various immune variables were assessed by univariate and multivariate linear regression analyses, adjusting for immuno-virologic and HIV specific factors as well as for risk factors for insulin resistance and diabetes. For soluble plasma biomarkers, biomarkers were examined by multivariate analysis if the univariate analysis produced a p<0.10. MO differences by diabetes categories and by presence or absence of metabolic syndrome were assessed by ANOVA and ANCOVA. All statistical analyses were conducted in SPSS (IBM, Version 21). A two-sided p-value < 0.05 was regarded as statistically significant.

## Results

### Patient Characteristics

A total of 141 subjects enrolled into the HAHC-CVD cohort who had available MO phenotype data were included in this analysis. The demographic, clinical, and immunologic characteristics of the subjects are summarized in Table 1. The majority of the subjects were male and of Caucasian ethnicity. Entry criteria for the cohort required subjects to be on ART and 86.5% of these subjects were virologically suppressed at a plasma HIV RNA level of < 50 copies/mL. The study recruited subjects age 40 or older, and the median age of the selected subjects in this dataset was 50 years. The median current CD4 count (percent) was relatively high at 505 cells/µL (29%) but the median self-reported nadir CD4 was relatively low at 150 cells/µL. A total of 22.3% of the population had evidence of glucose dysregulation. The median MO percent was 8%, and the median total MO count was 400 cells/µL. By subsets, the classical MO comprised the majority (76%) of the MO population with intermediate, non-classical and transitional MO comprising the rest in our gating strategy.

**Table 1:** Baseline characteristics of the subjects.

Clinical and Demographic Information	
Age	50 [45, 57]
Males, n (%)	125 (88.6%)
Race/ethnicity	
White	85 (60.3%)
Full or part Hawaiian/Pacific Islander (NH/PI)	18 (12.8%)
Asian	8 (5.7%)
African-American	5 (3.6%)
Native American/Alaskan	3 (2.1%)
Mixed (other than NH/PI)	22 (15.6%)
Hepatitis C, n (%)^a^	16 (11.3%)
Body mass index (kg/m^2^)	25.8 [23.7, 27.9]
Waist to hip ratio	
Male	0.94 [0.91, 0.98]
Female	0.94 [0.79, 0.95]
Systolic BP/diastolic BP	120 [113, 129]/75 [69, 82]
History of smoking, n (%)	93 (66.4%)
Glucose metabolism^b^	
Normal	109 (77.8%)
Impaired fasting glucose(IFG)/glucose tolerance(IGT)	16 (11.4%)
Diabetes	15 (10.7%)
Fasting glucose (mg/dL)	87 [81, 94]
HOMA-IR	1.49 [0.81, 2.50]
Total cholesterol (mg/dL)	177 [155, 202]
LDL cholesterol (mg/dL)	107 [89, 130]
HDL cholesterol (mg/dL)	42 [33, 53]
Triglyceride (mg/dL)	113 [82, 166]
Metabolic Syndrome n(%)^c^	25 (17.7%)
**Immunologic Parameters**	
WBC count (×10^9^ cells/L)	5.3 [4.3, 6.3]
Monocyte (%)	8 [7], [9]
% undetectable HIV RNA (< 50 copies/mL)	122 (86.5%)
Nadir CD4 count (cells/µL)^d^	150 [50, 245]
Current CD4 count (cells/µL)	505 [341, 640]
Current CD4%	29 [21], [36]
Current CD8%	44 [36, 52]
CD38+HLA-DR CD8 T-lymphocytes (%)	10.5 [7.7, 16.8]
CD38+HLA-DR CD8 T-lymphocytes (cells/µL)	76.6 [47.8, 124.1]
Monocyte Subsets (%)^e^	
Classical (CD14^++^CD16^−^)	76 [71.7, 81.9]
Intermediate (CD14^++^CD16^+^)	1.6 [0.60, 4.1]
Transitional CD14^+^CD16^−^)	14.1 [9.3, 18.9]
Non-classical (CD14^+/low^CD16^++^)	5.9 [4.0, 8.7]
Absolute monocyte count (cells/µL)	400 [329, 530]
Classical (CD14^++^CD16^−^)	310 [231, 410]
Intermediate (CD14^++^CD16^+^)	6.3 [2.5, 15.8]
Transitional CD14^+^CD16^−^)	55.8 [38.6, 81.3]
Non-classical (CD14^+/low^CD16^++^)	24.6 [13.7, 35.3]

All values reported are median [quartile1, quartile 3], except for frequency counts [n (%)].

HOMA-IR: Homeostatic Model Assessment of Insulin Resistance.

a By self-report, one patient with unknown HCV status.

b One patient with missing data.

c Based on the United States Cholesterol Education Program Adult Treatment Panel Guidelines (2001).

d Nadir CD4 count based on self-report (data available from 132 subjects).

e The sum may not necessarily add to 100% as the median percentage rather than the mean is reported.

### Relationship of CD4 and CD8 T cell Parameters to MO and Plasma Soluble Biomarkers

Self-reported nadir CD4 T cell count did not correlate with numbers of total MO or MO subsets. Similarly current CD4 T cell count as an absolute value did not correlate with any MO parameters. However, multiple correlations were seen between current CD4 T cells examined as a percentage of lymphocytes and various MO parameters. Current CD4 percent correlated negatively with log-total MO (r = −0.23, p = 0.006) and MO subsets log-classical MO (r = −0.19, p = 0.023) and log-non-classical MO (r = −0.23, p = 0.005) with a trend for log-transitional MO (r = −0.15, p = 0.080). No correlation was found between current CD4 percent and log-intermediate MO (r = 0.05, p = 0.561).

**Figure 2:**
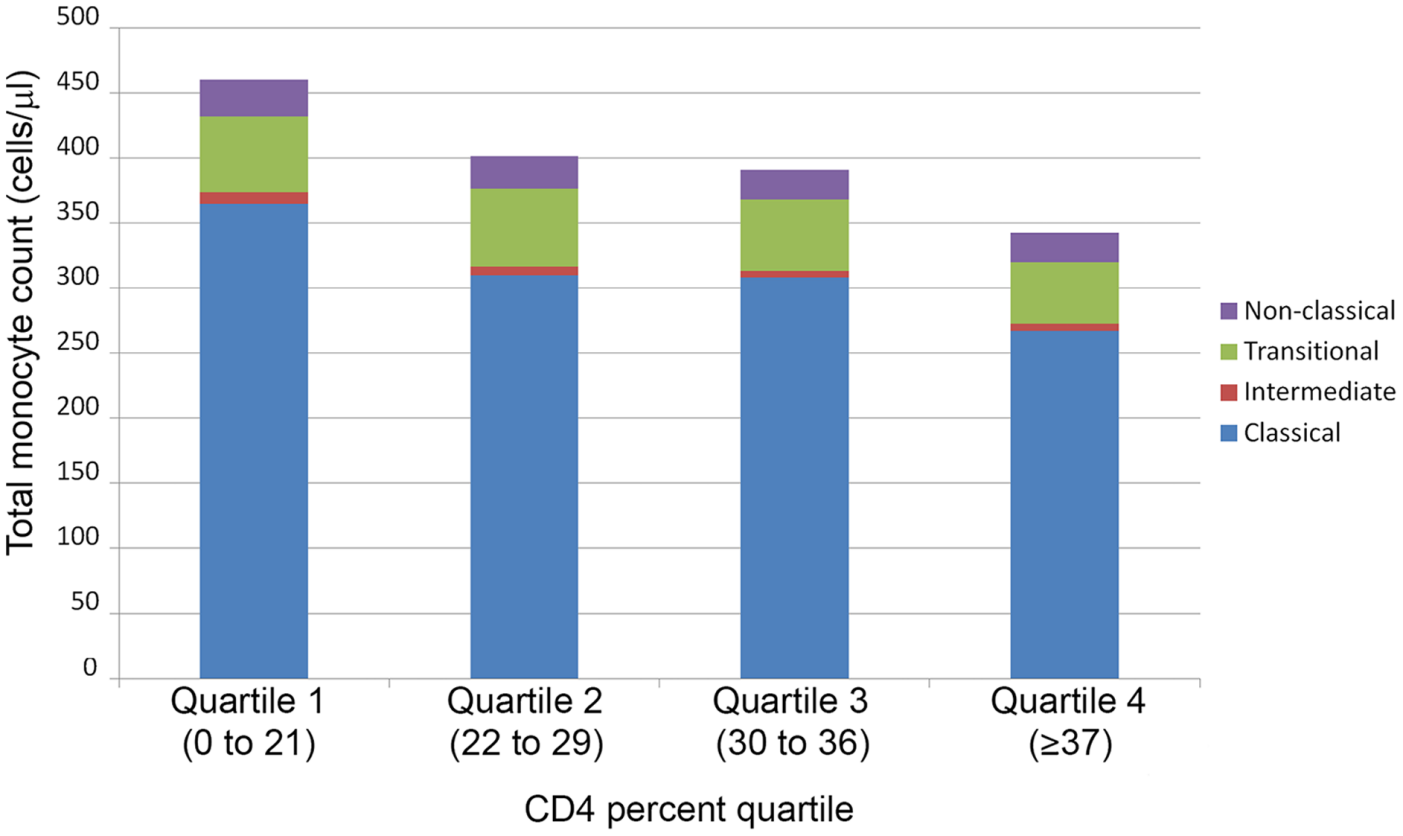
Absolute counts (cells/µL) of total monocytes by classical, intermediate, transitional and non-classical monocyte subset components by CD4 T cell percent quartiles.

The association between CD4 percent and MO can be seen visually in Figure 2 which shows the median total and subset MO numbers categorized by CD4 percent quartiles. Total MO increased in stepwise fashion with lower CD4 percent quartiles. All MO subsets contributed to this increase, although with a slightly higher contribution from non-classical MO. This could be demonstrated by the negative correlation between CD4 percent and percent (rather than the absolute value) of MO comprising non-classical MO (r = −0.19, p = 0.024).

Because decreases in CD4 percent can result not only from a decrease in the number of CD4 T cells but by an increase in other lymphocyte cell types including CD8 T cells, we assessed the relationship between CD4 percent and CD8 T cell parameters. CD4 percent correlated negatively with log-total CD8 T cells (r = −0.45, p<0.001) and with log-total CD38+HLA-DR+CD8 T cells (r = −0.49, p<0.001). In turn, log-total CD8 T cell count correlated positively with log-total MO (r = 0.35, p<0.001), log-classical MO (r = 0.27, p = 0.001), and log-transitional MO (r = 0.29, p = 0.001), with marginal significance with log-non-classical MO (r = 0.16, p = 0.061). Similar relationships were seen for activated CD8 T cells. Log-CD38^+^HLA-DR^+^CD8 T cells correlated with log-total MO (r = 0.36, p<0.001), log-classical MO (r = 0.29, p<0.001), log-transitional MO (r = 0.29, p = 0.001), and log-non-classical MO (r = 0.20, p = 0.018). No correlation was seen between log-CD38+HLA-DR+CD8 T cells and log-intermediate MO (r = −0.02, p = 0.81).

The database was examined for any correlations between CD4 percent and various soluble plasma biomarkers. Total CD4 count did not correlate with any soluble plasma biomarkers assessed. CD4 percent correlated negatively with log-transformed values of tPAI-1 (r = −0.19, p = 0.032) and TNF-α (r = −0.20, p = 0.028). Total CD8 T cell count correlated with log-sVCAM (r = 0.20, p = 0.026). CD8 T cell percent correlated with log-TNF-α (r = 0.19, p = 0.040).

### Relationship of Cellular and Soluble Plasma Biomarkers to HOMA-IR

**Figure 3:**
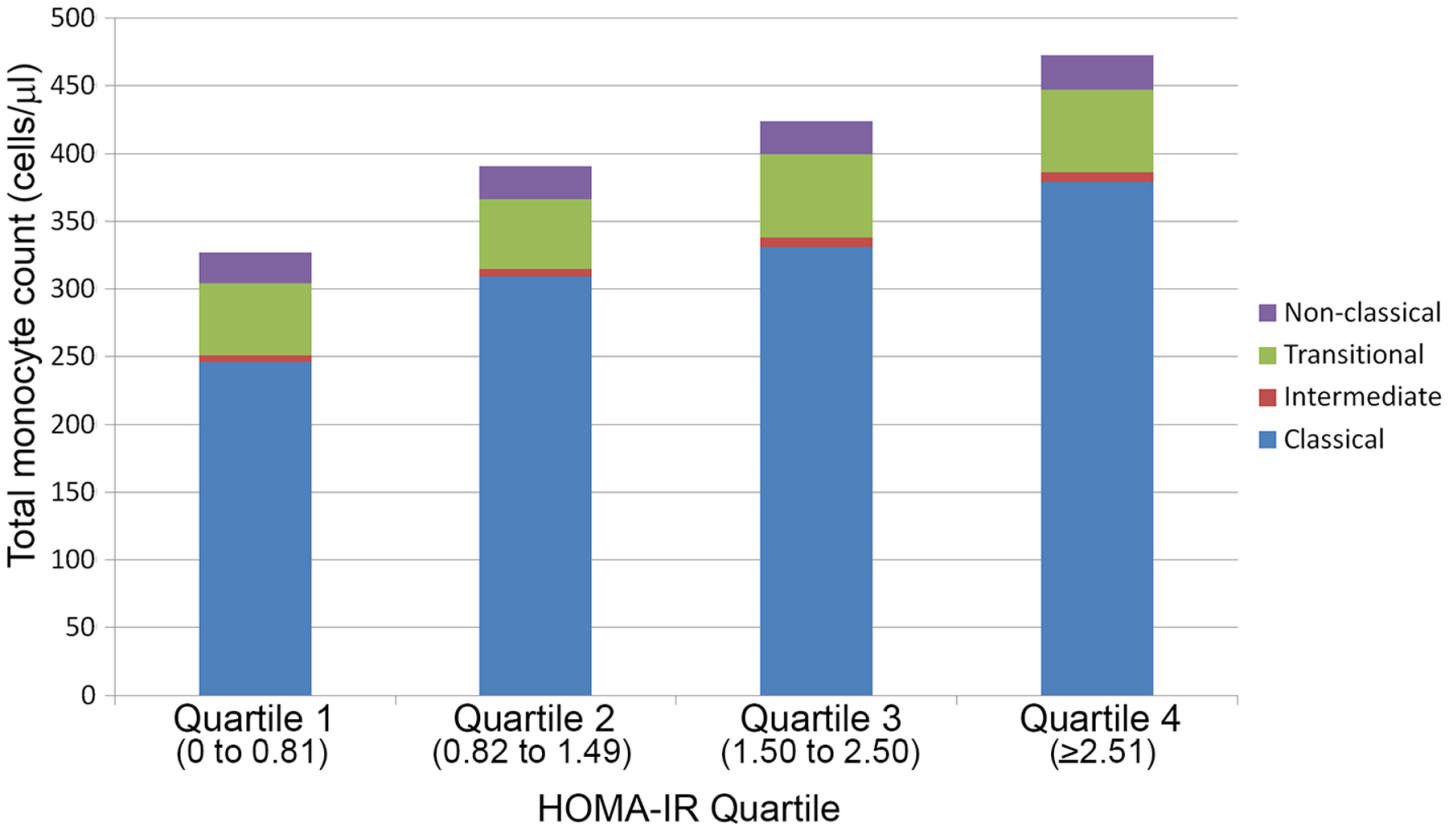
Absolute counts (cells/µL) of total monocytes by classical, intermediate, transitional and non-classical monocyte subset components by HOMA-IR quartiles.

No associations were seen between CD4 percent or log-total CD4 count and HOMA-IR (r = −0.08, p = 0.359 and r = 0.05, p = 0.581 respectively). Similarly, there was no association between CD8 T cell parameters (CD8 percent, log-total CD8 T cells, log-total CD38+HLA-DR+CD8 T cells) or log-CD4/CD8 ratio and HOMA-IR. However, log-total MO (r =  0.26, p = 0.002) and log-total classical MO (r = 0.26, p = 0.002) correlated positively with HOMA-IR. These associations are shown graphically in Figure 3 which depicts the quantity of total MO and its subsets by increasing HOMA-IR quartiles. The increase in total MO with higher HOMA-IR quartiles was contributed primarily by an increase in classical MO.

The relationship of MO to HOMA-IR was examined further in multivariate linear regression models with HOMA-IR as the dependent variable, adjusting for immuno-virologic and HIV specific factors (CD4 percent, presence/absence of plasma HIV RNA, and current protease inhibitor use) as well as for traditional risk factors for diabetes (age, gender, race, hepatitis C, smoking, body mass index [BMI]). Of these parameters, BMI (β = 0.03, p<0.001) and self-reported hepatitis C co-infection (β = 0.21, p = 0.021) were significantly associated in univariate linear regression with log-HOMA-IR. As shown in Table 2 using our multivariate model, log-total MO (β = 0.42, p = 0.018) remained a significant predictor of log-HOMA-IR. When this relationship was examined individually by specific MO subsets, log-classical MO was also a significant predictor (β = 0.35, p = 0.023), but not the other subsets.

**Table 2:** Multivariate linear regression analysis of the relative contribution of total monocyte count to HOMA-IR adjusting for various HIV-associated and diabetes risk factors.

	Regression coefficient	Standard Error	p-value	95% Confidence	Interval
Age, years	0.004	0.003	0.239	−0.003	0.011
Male (yes or no)	−0.028	0.086	0.743	−0.198	0.141
Caucasian (yes or no)	−0.021	0.055	0.710	−0.129	0.088
Hepatitis C (yes or no)	0.132	0.080	0.103	−0.027	0.290
Past or current smoking (yes or no)	−0.032	0.055	0.565	−0.140	0.077
Body Mass Index (kg/m^2^)	0.035	0.006	<0.001*	0.023	0.047
Current protease inhibitor use (yes or no)	−0.012	0.057	0.834	−0.124	0.100
Current CD4%	−0.001	0.003	0.788	−0.006	0.004
Plasma HIV RNA <50 copies/mL (yes or no)	0.036	0.080	0.652	−0.123	0.195
Log-total monocyte count	0.424	0.177	0.018*	0.073	0.775

*statistically significant.

Univariate linear regression was used to identify soluble plasma biomarkers associated with log-HOMA-IR to select biomarkers for testing in multivariate models. The following biomarkers were associated with log-HOMA-IR in a univariate model at p<0.10 level: sE-selectin, tPAI-1, CRP, SAP, IL-6, and IL-8. When examined by multivariate linear regression adjusting for immuno-virologic and cardio-metabolic parameters as performed for MO, log-tPAI-1 (β = 0.74, p<0.001) alone remained associated with log-HOMA-IR (Table 3).

**Table 3:** Multivariate linear regression analysis of the relative contribution of tissue plasminogen activator inhibitor-1 (tPAI-1) to HOMA-IR adjusting for various HIV-associated and diabetes risk factors

	Regression coefficient	Standard Error	p-value	95% Confidence	Interval
Age, years	0.008	0.003	0.019*	0.001	0.015
Male (yes or no)	−0.051	0.081	0.530	−0.211	0.109
Caucasian (yes or no)	−0.029	0.054	0.598	−0.136	0.079
Hepatitis C (yes or no)	0.164	0.085	0.055	−0.004	0.332
Past or current smoking (yes or no)	−0.035	0.054	0.514	−0.142	0.072
Body Mass Index, (kg/m^2^)	0.030	0.006	<0.001*	0.018	0.042
Current protease inhibitor use (yes or no)	−0.031	0.057	0.589	−0.143	0.082
Current CD4%	0.0003	0.002	0.895	−0.005	0.005
Plasma HIV RNA < 50 copies/mL (yes or no)	−0.019	0.082	0.812	−0.182	0.143
Log-tPAI-1	0.745	0.133	<0.001*	0.480	1.010

*statistically significant.

### Relationship of Cellular and Soluble Plasma Biomarkers to Fasting Glucose and Clinical Diabetes Classification

The only associations found between cellular biomarkers and fasting glucose values was that between log-total CD4 count and log-fasting glucose (r = 0.17, p = 0.043). This association was no longer present when adjusted for immuno-virologic and diabetes risk factors.

Log-transformed sE-selectin (β = 0.08, p = 0.007), MPO (β = 0.04, p = 0.089), tPAI-1 (β = 0.08, p = 0.050), IL-8 (β = 0.08, p = 0.053), and IL-10 (β = 0.02, p = 0.064) values were associated with log-fasting blood glucose at p<0.10 level using simple linear regression. Analyses by multivariate linear regression adjusting for immuno-virologic and diabetes risk parameters showed weak associations of uncertain significance [log-sE-selectin (β = 0.07, p = 0.044), log-tPAI-1 (β = 0.09, p = 0.035), and log-IL-10 (β = 0.02, p = 0.037)].

No differences by diabetes clinical classification (normal, IFG/IGT, or diabetes) were found for any cellular parameter except for total CD8 T cell count (cells/µL) [means(SD): normal 796.1 (361.8), IFG/IGT 1017.6 (590.2), diabetes: 661 (304.6), p = 0.034]. In terms of soluble biomarkers, differences by diabetes clinical classification were found for various biomarkers (pg/mL): sICAM [means(SD): normal 151.2 (61.0), IFG/IGT 242.5 (298.6), diabetes: 144.9 (117.0); p = 0.026], MPO [normal 19.0 (14.7), IFG/IGT: 16.3 (6.0), diabetes 44.1 (SD 81.6); p = 0.014], MCP-1 [normal 144.4 (51.6), IFG/IGT 146.7 (52.3), diabetes 186.8 (64.5); p = 0.045], and VEGF [normal 37.4 (34.6), IFG/IGT 36.8 (42.9), diabetes 79.7 (110.2); p = 0.019]. Comparisons by t-test showed that MPO (p = 0.004), MCP-1 (p = 0.013), and VEGF (p = 0.009) were significantly higher among diabetic subjects compared to subjects with normal glucose levels.

### Relationship of Immune Parameters to Metabolic Syndrome

No differences were found for individuals with or without metabolic syndrome by CD4 or CD8 parameters. Higher total MO levels were found in subjects with metabolic syndrome: [mean (SD): 504 (143) cells/µL in subjects with vs 427 (161) cells/µL in subjects without metabolic syndrome, p = 0.029]. This result remained significant when adjusted for age, gender and ethnicity (p = 0.013). By subsets, a trend towards significance was seen for difference in classical MO (p = 0.051) and for intermediate MO (p = 0.059). Soluble biomarkers sE-selectin (p = 0.007) and sICAM (p = 0.041) were higher in subjects with metabolic syndrome.

### Correlations between Monocytes and Activated CD8 T Cells and Soluble Plasma Biomarkers

The correlations between MO and activated CD8 T cells and soluble plasma biomarkers are shown in Table 4. Correlations at p<0.01 were seen between CD38^+^HLA-DR^+^CD8 T cells and sICAM and TNF-α. A larger number of positive correlations were found between MO and/or MO subsets and various plasma biomarkers. Specifically, correlations at p<0.01 were found between total MO and CRP, IL-6, MMP-9, MPO, SAA and tPAI-1. Associations between classical MO and intermediate MO and various biomarkers overall mirrored the correlations seen with total MO. Non-classical MO showed correlations at p<0.01 with MCP-1 and SAP.

**Table 4:** Correlation table of absolute counts of total monocytes, monocyte subsets and CD38^+^HLA-DR^+^CD8 T cells with various plasma cytokines.

	Total monocyte count	Classical CD14^++^CD16^−^	Intermediate CD14^++^CD16^+^	Transitional CD14^+^CD16^−^	Non-classical CD14^+/low^CD16^++^	CD38^+^HLA-DR^+^ CD8 T cells
CRP	0.23**	0.27**	0.47**	−0.10	0.15	−0.09
IFN-γ	0.02	−0.06	0.10	0.12	0.01	0.13
IL-1β	0.22*	0.18	−0.06	0.20*	0.15	0.02
IL-6	0.29**	0.35**	0.20*	0.03	0.04	0.03
IL-8	0.16	0.16	0.05	0.04	−0.05	−0.10
IL-10	0.21*	0.24**	0.05	0.10	0.09	0.20*
MCP-1	0.14	0.12	0.21*	−0.05	0.29**	0.10
MMP-9	0.30**	0.30**	0.31**	0.08	0.05	−0.02
MPO	0.29**	0.30**	0.42**	−0.01	0.14	0.18*
NT-proBNP	0.16	0.12	−0.02	0.14	0.04	0.12
SAA	0.29**	0.31**	0.43**	−0.05	0.23*	0.07
SAP	0.17	0.18*	0.45**	−0.17	0.26**	−.04
sE-selectin	0.11	0.11	0.22*	−0.07	0.09	−.08
sICAM-1	0.08	0.11	0.10	−0.07	−0.06	0.25**
sVCAM-1	0.08	0.10	0.27**	−0.12	0.19*	0.18*
TNF-α	0.19*	0.17	−0.06	0.16	0.03	0.29**
tPAI-1	0.32**	0.28**	0.18*	0.20*	0.12	0.003
VEGF	0.13	0.13	−0.02	0.23*	−0.08	0.02

Values were log-transformed prior to analysis.

Pearson Correlation was utilized.

*Correlation significant at the 0.05 level (2-tailed).

** Correlation significant at the 0.01 level (2-tailed).

## DISCUSSION

Our study found that HIV-associated immune dysregulation, as assessed by a decrease in CD4 percent is characterized by an increase in the total MO population with a small rise in the percentage accounted for by non-classical MO. This association was seen specifically with CD4 T cells assessed as a percentage of CD4 T cells and not as an absolute value of total CD4 T cells. In turn, we found that an increase in total MO, comprised largely of classical MO, accompanies insulin resistance. This association remained after adjustment for HIV immuno-virologic and traditional diabetes risk factors. Higher levels of MO were seen in subjects with metabolic syndrome. Positive correlations were detected between MO and many plasma biomarkers. Included among these biomarkers was tPAI-1 which also correlated inversely with CD4 percent, positively with MO levels, and predicted insulin resistance.

Insulin resistance is increasingly recognized as a state of chronic, low-level inflammation [22], [23], [24]. Immune activation and inflammation also occur in the context of HIV and are predictors of HIV disease progression and mortality, and of multiple non-infectious chronic complications [25], [26], [27]. Such activation may be secondary to HIV itself, to co-pathogens such as CMV or hepatitis C, or be secondary to microbial translocation across a damaged gut mucosa [28]. There is also increasing recognition that in addition to CD8 T cells, MO may play a role in the immune activation and inflammation that drive many of these disease processes [29].

Our study found an association between low CD4 percent and MO increase and, in turn MO increase and increase in insulin resistance. One potential interpretation of the data is that an increase in MO is part of HIV-induced immune activation, and that such increase in turn may drive the increased risk of insulin resistance seen in chronic HIV. While our study did not show a direct association between CD4 count, either as a percent or as total numbers, and insulin resistance or diabetes as shown in larger epidemiologic studies [2], [33], [34], [35], it is consistent with a study that demonstrated a higher prevalence of elevated sCD14, a marker of MO activation, among HIV-infected veterans with a CD4 cell count <200 cells/µL [33]. Our inability to find a direct link between CD4 percent and insulin resistance may be related to the small sample size of our cohort.

By subsets, classical, non-classical and transitional MO, but not intermediate MO, contributed to the increase in MO with falling CD4 percent. Non-classical MO represented a slightly larger percentage of this MO increase. The association between CD4 T cells and MO was specifically related to CD4 percent and not to total numbers of CD4 T cells. Decrease in CD4 percent may represent a decrease in total CD4 count or, may also be secondary to increase(s) in other lymphocyte subsets including CD8 T cells. Our study did find a negative correlation between CD4 percent and total numbers of CD8 T cells. However, neither total CD8 T cells nor total activated CD8 T cells explained insulin resistance, while a clear association was seen between total MO or classical MO and insulin resistance in adjusted models. It is worthy of note that a number of studies in the literature have found independent predictive values for percentage of CD4 T cells and CD4 absolute count in respect to HIV disease progression or death [30], [31], [32]. One study explored the role of CD8 T cells as a potential explanation for the association between CD4 percent and disease progression and found that it did not explain this relationship [31]. We speculate that CD4 percent may provide additional information on increased risk for cardio-metabolic complications mediated in part through MO immune activation. Analysis of large cohort datasets to determine whether CD4 percent and MO levels are related to such complications might be considered.

The association between MO and glucose metabolism identified in our study was primarily with insulin resistance with little or no relationship to fasting glucose levels or to diabetes classification. When examined by subsets, we found that classical MO, the largest subset represented, played the most prominent role in the MO increase associated with insulin resistance.

Metabolic syndrome is a constellation of inter-related metabolic risk factors associated with increased risk for the development of diabetes and cardiovascular disease. While its limitations and significance in the context of HIV is uncertain, the metabolic syndrome is closely related to the presence of insulin resistance, and, given the association between MO and insulin resistance found in our study, the finding of increased MO with metabolic syndrome is not surprising, and adds to the likelihood that MO do indeed play a pathologic role in increasing both diabetes and cardiovascular disease risk in the HIV-infected population.

Our study found multiple positive correlations between MO and/or its subsets and plasma biomarkers CRP, IL-6, MMP-9, MPO, SAA, SAP and tPAI-1. Significant correlations were also found between CD38^+^HLA-DR^+^CD8 T cells and biomarkers sICAM and TNF-α. Interestingly we also found correlations between low CD4 percent and TNF-α and tPAI-1, and correlations between HOMA-IR and CRP, IL-6, SAP, and tPAI-1. This suggests that cellular immune parameters and soluble plasma biomarkers are likely inter-related in the roles they play in HIV immune dysregulation and insulin resistance. These results are furthermore consistent with studies that have found higher levels of hs-CRP and sTNF1 and sTNF2 in association with insulin resistance and diabetes in the context of HIV [3], [36].

The associations found for tPAI-1 are worthy of particular attention. TPAI-1 is an inhibitor of fibrinolysis and believed to play a crucial role in the development of atherothrombotic disease. In our study, higher levels of tPAI-1 were associated with lower CD4 percent and predicted insulin resistance in an adjusted model. Our findings are consistent with studies in the general population that have demonstrated associations between high levels of circulating PAI-1 and insulin resistance, diabetes and cardiovascular disease [37], [38]. Levels of PAI-1 have been reported to be higher in HIV infected individuals than in seronegative controls [39], and to be associated with insulin resistance [40]. It has been linked to protease inhibitor-containing ART [41], and, in contrast to some other plasma markers of inflammation, have been reported to not improve after the initiation of ART [42]. Our finding of a negative correlation between tPAI-1 and CD4 percent suggests that in addition to the potential role of antiretroviral medications, HIV-associated immune dysregulation may play a role in high tPAI-1 levels seen in the treated population. Furthermore our study links tPAI-1 with total and classical MO but not with T cell activation.

This study is limited by its cross sectional nature and the relatively modest size of the cohort. However the strengths of the study are the careful clinical and metabolic characterization performed in the cohort in association with detailed phenotypic characterization of MO and CD8 T cells, and biomarker assays performed in plasma. We conclude that chronic HIV in subjects on stable ART is characterized not only by dysregulation and immune activation of CD8 T cells but also of MO, and that higher levels of MO may contribute to the increased rates of insulin resistance and metabolic syndrome seen in this population. MO may therefore play a substantial role in the increased risk of cardio-metabolic disease during chronic HIV. CD4 percent may provide additional information beyond absolute values about MO and metabolic risk in the HIV-infected population. Increase in MO is associated with increase in multiple plasma soluble biomarkers, including tPAI-1 which also predicts insulin resistance in adjusted models.

## References

[1] Gutierrez AD, Balasubramanyam A (2012) Dysregulation of glucose metabolism in HIV patients: epidemiology, mechanisms, and management. Endocrine 41: 1–10. 22134974 10.1007/s12020-011-9565-z PMC3417129

[2] Boufassa F, Goujard C, Viard JP, Carlier R, Lefebvre B, et al (2012) Immune deficiency could be an early risk factor for altered insulin sensitivity in antiretroviral-naive HIV-1-infected patients: the ANRS COPANA cohort. Antivir Ther 17: 91–100. 22267473 10.3851/IMP1916 PMC3893638

[3] Brown TT, Tassiopoulos K, Bosch RJ, Shikuma C, McComsey GA (2010) Association between systemic inflammation and incident diabetes in HIV-infected patients after initiation of antiretroviral therapy. Diabetes Care 33: 2244–2249. 20664016 10.2337/dc10-0633 PMC2945167

[4] Ziegler-Heitbrock L, Ancuta P, Crowe S, Dalod M, Grau V, et al (2010) Nomenclature of monocytes and dendritic cells in blood. Blood 116: e74–80. 20628149 10.1182/blood-2010-02-258558

[5] Jalbert E, Crawford TQ, D'Antoni ML, Keating SM, Norris PJ, et al (2013) IL-1Beta Enriched Monocytes Mount Massive IL-6 Responses to Common Inflammatory Triggers among Chronically HIV-1 Infected Adults on Stable Anti-Retroviral Therapy at Risk for Cardiovascular Disease. PLoS One 8: e75500. 24086545 10.1371/journal.pone.0075500 PMC3783392

[6] Barbour JD, Jalbert EC, Chow DC, Gangcuangco LM, Norris PJ, et al (2014) Reduced CD14 expression on classical monocytes and vascular endothelial adhesion markers independently associate with carotid artery intima media thickness in chronically HIV-1 infected adults on virologically suppressive anti-retroviral therapy. Atherosclerosis 232: 52–58. 24401216 10.1016/j.atherosclerosis.2013.10.021 PMC3919042

[7] Gordon S, Taylor PR (2005) Monocyte and macrophage heterogeneity. Nat Rev Immunol 5: 953–964. 16322748 10.1038/nri1733

[8] Strauss-Ayali D, Conrad SM, Mosser DM (2007) Monocyte subpopulations and their differentiation patterns during infection. J Leukoc Biol 82: 244–252. 17475785 10.1189/jlb.0307191

[9] Geissmann F, Jung S, Littman DR (2003) Blood monocytes consist of two principal subsets with distinct migratory properties. Immunity 19: 71–82. 12871640 10.1016/s1074-7613(03)00174-2

[10] Ziegler-Heitbrock L (2007) The CD14+ CD16+ blood monocytes: their role in infection and inflammation. J Leukoc Biol 81: 584–592. 17135573 10.1189/jlb.0806510

[11] Seidler S, Zimmermann HW, Bartneck M, Trautwein C, Tacke F (2010) Age-dependent alterations of monocyte subsets and monocyte-related chemokine pathways in healthy adults. BMC Immunol 11: 30. 20565954 10.1186/1471-2172-11-30 PMC2910032

[12] Ellery PJ, Tippett E, Chiu YL, Paukovics G, Cameron PU, et al (2007) The CD16+ monocyte subset is more permissive to infection and preferentially harbors HIV-1 in vivo. J Immunol 178: 6581–6589. 17475889 10.4049/jimmunol.178.10.6581

[13] Kusao I, Shiramizu B, Liang CY, Grove J, Agsalda M, et al (2012) Cognitive performance related to HIV-1-infected monocytes. J Neuropsychiatry Clin Neurosci 24: 71–80. 22450616 10.1176/appi.neuropsych.11050109 PMC3335340

[14] Funderburg NT, Zidar DA, Shive C, Lioi A, Mudd J, et al (2012) Shared monocyte subset phenotypes in HIV-1 infection and in uninfected subjects with acute coronary syndrome. Blood 120: 4599–4608. 23065151 10.1182/blood-2012-05-433946 PMC3512236

[15] Han J, Wang B, Han N, Zhao Y, Song C, et al (2009) CD14(high)CD16(+) rather than CD14(low)CD16(+) monocytes correlate with disease progression in chronic HIV-infected patients. J Acquir Immune Defic Syndr 52: 553–559. 19950429 10.1097/qai.0b013e3181c1d4fe

[16] Min D, Brooks B, Wong J, Salomon R, Bao W, et al (2012) Alterations in monocyte CD16 in association with diabetes complications. Mediators Inflamm 2012: 649083. 23316106 10.1155/2012/649083 PMC3536440

[17] Corrales JJ, Almeida M, Burgo RM, Hernandez P, Miralles JM, et al (2007) Decreased production of inflammatory cytokines by circulating monocytes and dendritic cells in type 2 diabetic men with atherosclerotic complications. J Diabetes Complications 21: 41–49. 17189873 10.1016/j.jdiacomp.2005.09.006

[18] Pandzic Jaksic V, Gizdic B, Miletic Z, Trutin-Ostovic K, Jaksic O (2013) Association of monocyte CCR2 expression with obesity and insulin resistance in postmenopausal women. Clin Invest Med 36: E24–31. 23374597 10.25011/cim.v36i1.19402

[19] Yang M, Gan H, Shen Q, Tang W, Du X, et al (2012) Proinflammatory CD14+CD16+ monocytes are associated with microinflammation in patients with type 2 diabetes mellitus and diabetic nephropathy uremia. Inflammation 35: 388–396. 21847775 10.1007/s10753-011-9374-9

[20] Executive summary: standards of medical care in diabetes—2013. Diabetes Care 36 Suppl 1S4–S10. 23264424 10.2337/dc13-S004 PMC3537272

[21] Grundy SM, Brewer HB Jr, Cleeman JI, Smith SC Jr, Lenfant C (2004) Definition of metabolic syndrome: Report of the National Heart, Lung, and Blood Institute/American Heart Association conference on scientific issues related to definition. Circulation 109: 433–438. 14744958 10.1161/01.CIR.0000111245.75752.C6

[22] Scarpellini E, Tack J (2012) Obesity and metabolic syndrome: an inflammatory condition. Dig Dis 30: 148–153. 22722429 10.1159/000336664

[23] Skalicky J, Muzakova V, Kandar R, Meloun M, Rousar T, et al (2008) Evaluation of oxidative stress and inflammation in obese adults with metabolic syndrome. Clin Chem Lab Med 46: 499–505. 18298345 10.1515/CCLM.2008.096

[24] Fernandez-Real JM, Ricart W (2003) Insulin resistance and chronic cardiovascular inflammatory syndrome. Endocr Rev 24: 278–301. 12788800 10.1210/er.2002-0010

[25] Kuller LH, Tracy R, Belloso W, De Wit S, Drummond F, et al (2008) Inflammatory and coagulation biomarkers and mortality in patients with HIV infection. PLoS Med 5: e203. 18942885 10.1371/journal.pmed.0050203 PMC2570418

[26] Sandler NG, Wand H, Roque A, Law M, Nason MC, et al (2011) Plasma levels of soluble CD14 independently predict mortality in HIV infection. J Infect Dis 203: 780–790. 21252259 10.1093/infdis/jiq118 PMC3071127

[27] Deeks SG, Phillips AN (2009) HIV infection, antiretroviral treatment, ageing, and non-AIDS related morbidity. BMJ 338: a3172. 19171560 10.1136/bmj.a3172

[28] Deeks SG (2011) HIV infection, inflammation, immunosenescence, and aging. Annu Rev Med 62: 141–155. 21090961 10.1146/annurev-med-042909-093756 PMC3759035

[29] Jalbert E CT, D'Antoni ML, Keating SM, Norris PJ, Nakamoto BK, et al.. (2013) IL-1b Enriched Monocytes Mount Massive IL-6 Responses to Common Inflammatory Triggers among Chronically HIV-1 Infected Adults on Stable Anti-Retroviral Therapy at Risk for Cardiovascular Disease. PLoS ONE in press. 10.1371/journal.pone.0075500 PMC3783392 24086545

[30] Hulgan T, Raffanti S, Kheshti A, Blackwell RB, Rebeiro PF, et al (2005) CD4 lymphocyte percentage predicts disease progression in HIV-infected patients initiating highly active antiretroviral therapy with CD4 lymphocyte counts >350 lymphocytes/mm3. J Infect Dis 192: 950–957. 16107946 10.1086/432955

[31] Hulgan T, Shepherd BE, Raffanti SP, Fusco JS, Beckerman R, et al (2007) Absolute count and percentage of CD4+ lymphocytes are independent predictors of disease progression in HIV-infected persons initiating highly active antiretroviral therapy. J Infect Dis 195: 425–431. 17205482 10.1086/510536

[32] Moore DM, Hogg RS, Yip B, Craib K, Wood E, et al (2006) CD4 percentage is an independent predictor of survival in patients starting antiretroviral therapy with absolute CD4 cell counts between 200 and 350 cells/microL. HIV Med 7: 383–388. 16903983 10.1111/j.1468-1293.2006.00397.x

[33] Armah KA, McGinnis K, Baker J, Gibert C, Butt AA, et al (2012) HIV status, burden of comorbid disease, and biomarkers of inflammation, altered coagulation, and monocyte activation. Clin Infect Dis 55: 126–136. 22534147 10.1093/cid/cis406 PMC3493182

[34] El-Sadr WM, Mullin CM, Carr A, Gibert C, Rappoport C, et al (2005) Effects of HIV disease on lipid, glucose and insulin levels: results from a large antiretroviral-naive cohort. HIV Med 6: 114–121. 15807717 10.1111/j.1468-1293.2005.00273.x

[35] Petoumenos K, Worm SW, Fontas E, Weber R, De Wit S, et al (2012) Predicting the short-term risk of diabetes in HIV-positive patients: the Data Collection on Adverse Events of Anti-HIV Drugs (D:A:D) study. J Int AIDS Soc 15: 17426. 23078769 10.7448/IAS.15.2.17426 PMC3494158

[36] Limone P, Biglino A, Valle M, Degioanni M, Paola Servato M, et al (2003) Insulin resistance in HIV-infected patients: relationship with pro-inflammatory cytokines released by peripheral leukocytes. J Infect 47: 52–58. 12850163 10.1016/s0163-4453(03)00055-0

[37] Hamsten A, Wiman B, de Faire U, Blomback M (1985) Increased plasma levels of a rapid inhibitor of tissue plasminogen activator in young survivors of myocardial infarction. N Engl J Med 313: 1557–1563. 3934538 10.1056/NEJM198512193132501

[38] Erzen B, Sabovic M (2013) In young post-myocardial infarction male patients elevated plasminogen activator inhibitor-1 correlates with insulin resistance and endothelial dysfunction. Heart Vessels 28: 570–577. 23001714 10.1007/s00380-012-0287-9

[39] Guzman-Fulgencio M, Medrano J, Rallon N, Echeverria-Urabayen A, Miguel Benito J, et al (2011) Soluble markers of inflammation are associated with Framingham scores in HIV-infected patients on suppressive antiretroviral therapy. J Infect 63: 382–390. 21855573 10.1016/j.jinf.2011.08.006

[40] De Larranaga G, Galich A, Puga L, Alonso B, Benetucci J (2004) Insulin resistance status is an important determinant of PAI-1 levels in HIV-infected patients, independently of the lipid profile. J Thromb Haemost 2: 532–534. 15009482 10.1111/j.1538-7836.2004.00602.x

[41] Koppel K, Bratt G, Schulman S, Bylund H, Sandstrom E (2002) Hypofibrinolytic state in HIV-1-infected patients treated with protease inhibitor-containing highly active antiretroviral therapy. J Acquir Immune Defic Syndr 29: 441–449. 11981359 10.1097/00042560-200204150-00003

[42] van Vonderen MG, Hassink EA, van Agtmael MA, Stehouwer CD, Danner SA, et al (2009) Increase in carotid artery intima-media thickness and arterial stiffness but improvement in several markers of endothelial function after initiation of antiretroviral therapy. J Infect Dis 199: 1186–1194. 19275490 10.1086/597475

